# Voltage-Dependent Regulation of Complex II Energized Mitochondrial Oxygen Flux

**DOI:** 10.1371/journal.pone.0154982

**Published:** 2016-05-06

**Authors:** Fan Bai, Brian D. Fink, Liping Yu, William I. Sivitz

**Affiliations:** 1 Department of Biochemistry, University of Iowa, Iowa City, IA, 52242, United States of America; 2 Department of Internal Medicine / Endocrinology and Metabolism, University of Iowa and the Iowa City Veterans Affairs Medical Center, Iowa City, IA, 52242, United States of America; 3 NMR Core Facility, University of Iowa, Iowa City, IA, 52242, United States of America; University of Nebraska Medical Center, UNITED STATES

## Abstract

Oxygen consumption by isolated mitochondria is generally measured during state 4 respiration (no ATP production) or state 3 (maximal ATP production at high ADP availability). However, mitochondria *in vivo* do not function at either extreme. Here we used ADP recycling methodology to assess muscle mitochondrial function over intermediate clamped ADP concentrations. In so doing, we uncovered a previously unrecognized biphasic respiratory pattern wherein O_2_ flux on the complex II substrate, succinate, initially increased and peaked over low clamped ADP concentrations then decreased markedly at higher clamped concentrations. Mechanistic studies revealed no evidence that the observed changes in O_2_ flux were due to altered opening or function of the mitochondrial permeability transition pore or to changes in reactive oxygen. Based on metabolite and functional metabolic data, we propose a multifactorial mechanism that consists of coordinate changes that follow from reduced membrane potential (as the ADP concentration in increased). These changes include altered directional electron flow, altered NADH/NAD^+^ redox cycling, metabolite exit, and OAA inhibition of succinate dehydrogenase. In summary, we report a previously unrecognized pattern for complex II energized O_2_ flux. Moreover, our findings suggest that the ADP recycling approach might be more widely adapted for mitochondrial studies.

## Introduction

Functional studies of isolated mitochondria dating several decades have usually been carried out in the absence of ADP (herein considered as state 4 respiration, acknowledging that some would label as state 2) or presence of ADP (state 3) [1]. In many cases, investigators have used the data to calculate parameters including the respiratory control ratio (state 3/state 4 respiration) or the amount of ADP consumed per amount of oxygen utilized during state 3 (ADP to O ratio) [1]. Such studies have been widely applied to describe mitochondrial function as affected by a myriad of physiologic or pathophysiologic states. However, these studies are confounded since, under physiologic conditions, mitochondria do not function at either state 3 or 4, but rather in between. Moreover, when ADP is added to measure state 3 respiration, the nucleotide is usually added in amounts that induce near maximal O_2_ flux until nearly all is consumed. Thereafter, respiration quickly returns to state 4, leaving no indication of mitochondrial function under actual intermediate respiratory conditions.

Mitochondrial function over intermediate respiratory states has been assessed in the past by different methods designed to regenerate ADP for phosphorylation. These include the use of creatine plus creatine kinase [2–4], ATPase with excess ATP [2, 5–7], and glucose plus hexokinase [4, 8]. Most of these studies were directed at liver mitochondria and used for kinetic analysis to determine flux control parameters and not broadly applied to physiologic conditions.

Here we used ADP recycling technology to assess mouse skeletal muscle mitochondrial function over respiratory states ranging between 4 and 3. In so doing, we uncovered a previously unreported biphasic respiratory pattern regarding respiration on the complex II substrate, succinate (without rotenone), as a function of added ADP. As we could find no prior recognition of this phenomenon, we carried out several studies to assess the underlying mechanism.

## Materials and Methods

### Reagents and supplies

Reagents, kits, and supplies were as specified or purchased from standard sources.

### Animal procedures

Mice were maintained according to National Institute of Health guidelines and the protocol was approved by our Iowa City Veterans Affairs Medical Center Institutional Animal Care and Use Committee. Mice were monitored daily for any abnormality in movement, feeding, or distressed appearance. No animal became sick or died prior to the experimental endpoint. Mice were euthanized by isoflurane inhalation followed by thoracotomy and cardiac puncture.

Mice deficient in the critical cyclophilin D (CypD) component of the mitochondrial permeability transition pore (MTP) (CypD^null^) and littermate controls were purchased from Jackson Laboratories, Bar Harbor, ME. The phenotype of the mutant mouse is overtly normal [9], but mitochondria from these mice have an increased capacity to retain calcium and CypD-deficient mouse embryonic fibroblasts (MEFs) are resistant to oxidative stress-induced cell death compared to WT MEFs [9]. Mice were fed a normal rodent diet (13% kcal fat) (diet 7001, Teklad, Harlan Labs, Madison, WI) until sacrifice at age 6 to 8 weeks.

### Preparation of mitochondria

Total hind limb muscle, liver, heart, or brain mitochondria were prepared by differential centrifugation and purification on a Percoll gradient as we have described in the past [10]. Mitochondrial integrity was assessed by cytochrome C release using a commercial kit (Cytochrome C Oxidase Assay Kit, Sigma-Aldrich, St. Louis) indicating a mean of 96% intact mitochondria over three assays, well within an acceptable range compared to mitochondrial preparations from several sources [11].

### ADP recycling and generation of the 2-deoxyglucose ATP energy clamp

**Fig 1:**
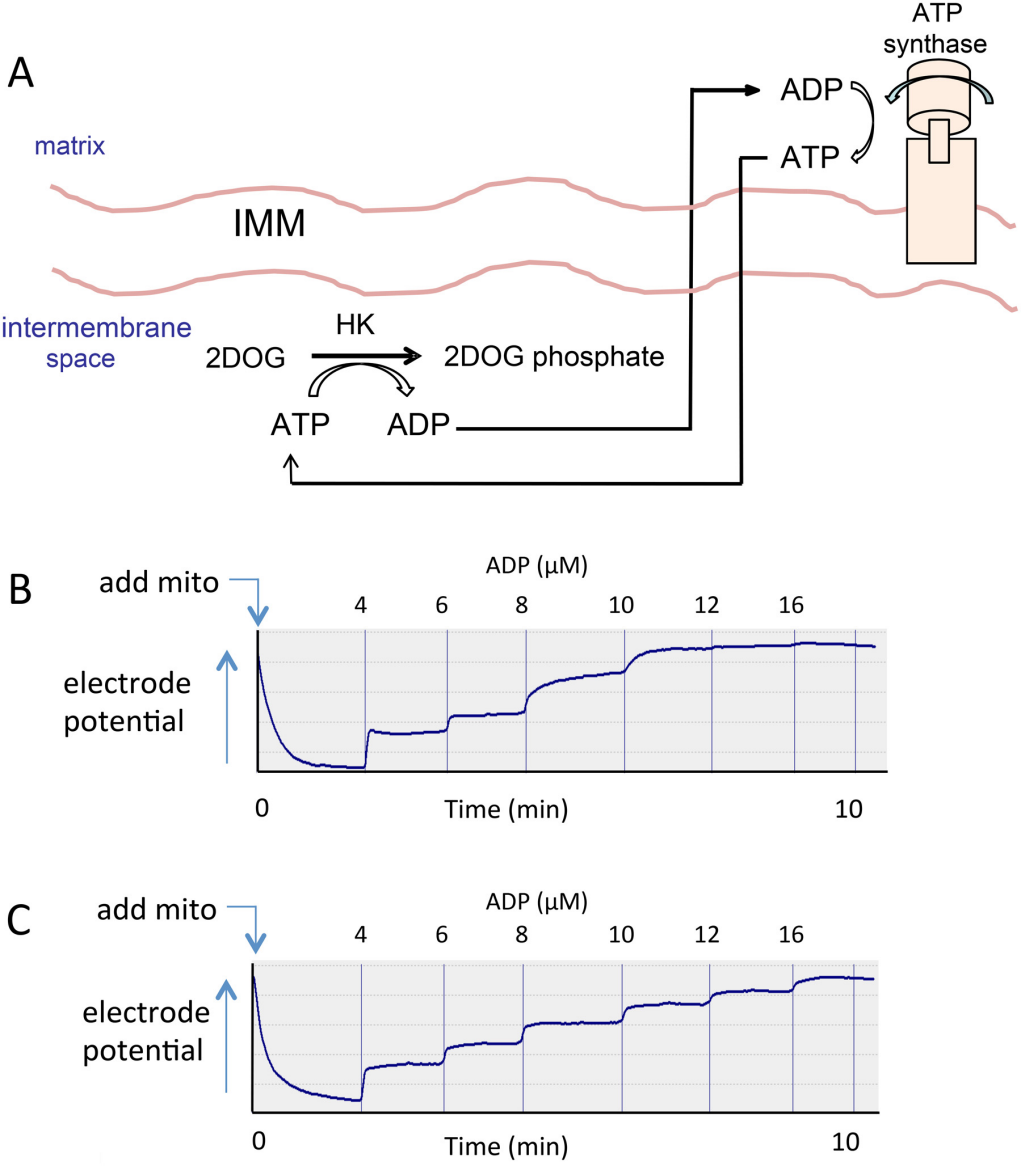
The 2-deoxyglucose (2DOG) energy clamp. (A) Graphic depiction. Saturating amounts of 2-deoxyglucose (2DOG) and hexokinase (HK) recycle ATP back to ADP by rapidly and irreversibly converting 2DOG into 2-deoxyglucose phosphate (2DOGP). The ADP concentration is clamped at levels determined by the amount of ADP added. IMM = inner mitochondrial membrane. (B and C) Oxygraph tracings of inner membrane potential (inversely related to electrode potential shown on y-axis after calculation using the Nernst equation) vs. time obtained by incubating mouse hindlimb muscle mitochondria (mito), 0.05 mg/ml, fueled by 5 mM succinate (panel B) or 5 mM succinate plus 5 μM rotenone (panel C). ADP was added in incremental amounts to generate the final recycling nucleotide phosphate concentrations shown. After each addition, plateau values were reached, consistent with recycling at a steady [ADP].

We used a method that we recently developed [12] to carry out bioenergetic studies of isolated mitochondria under conditions of clamped ADP and membrane potential (ΔΨ). Studies were carried out in the presence of hexokinase (HK) and excess 2DOG and varying amounts of added ADP. ATP generated from ADP under these conditions drives the conversion of 2DOG to 2DOG phosphate (2DOGP) while regenerating ADP (Fig 1A). The reaction occurs rapidly and irreversibly, thereby effectively clamping ADP concentrations and, consequently, also clamping ΔΨ and the respiratory state dependent on the amount of exogenous ADP added. This was in fact the case (Fig 1B and 1C), as we have demonstrated in the past for rat muscle [12], mouse liver [13] and mouse heart [13] mitochondria.

We also assessed respiration while titrating with carbonyl cyanide p-[trifluoromethoxy]-phenyl-hydrazone (FCCP). This was done in order to determine the effect of perturbed ΔΨ *per se*, i.e. apart from the effect of ADP.

### Respiration and membrane potential

Respiration and ΔΨ were simultaneously determined using an Oxygraph-2k high resolution respirometer (Oroboros Instruments, Innsbruck, Austria) fitted with a potential sensitive tetraphenylphosphonium (TPP^+^) electrode. Mitochondria (0.05 mg/ml) were incubated at 37°C in 2 ml of ionic respiratory buffer (105 mM KCl, 10 mM NaCl, 5 mM Na_2_HPO_4_, 2 mM MgCl_2_, 10 mM HEPES pH 7.2, 1 mM EGTA, 0.2% defatted BSA) with 5 U/ml hexokinase (Worthington Biochemical), and 5 mM 2-deoxyglucose. ADP was added sequentially to achieve the desired final concentrations with plateaus in respiration and potential achieved after each addition. A TPP^+^ standard curve was performed in each run by adding tetraphenylphosphonium chloride at concentrations of 0.25, 0.5, 0.75, and 1 μM prior to the addition of mitochondria to the chamber.

### Use of the 2DOG ATP energy clamp to quantify ATP production in isolated mitochondria and simultaneous assessment of H_2_O_2_ production

Mitochondria (0.1 mg/ml) were added to individual wells of black polystyrene 96-well round bottom plates in a total volume of 60 μl and incubated at 37°C in respiratory buffer plus 5 units/ml HK (Worthington Biochemical) and 5 mM 2DOG in the presence of selected concentrations of ADP. After incubation for 20 min with gentle rocking, the contents of the microplate wells were removed to tubes on ice containing 1 μl of 120 μM oligomycin to inhibit ATP synthase. Tubes were then centrifuged for 4 minutes at 14,000 x g to pellet the mitochondria. Supernatants were transferred to new tubes and stored at -20°C for quantification of 2DOGP by nuclear magnetic resonance (NMR) spectroscopy. To prepare the NMR sample, 40 μl of assay supernatant was added to a 5 mm (OD) standard NMR tube (Norell, Inc.) along with 50 μl of deuterium oxide (D_2_O) and 390 μl of a buffer consisting of 120 mM KCl, 5 mM KH_2_PO_4_ and 2 mM MgCl_2_, pH 7.2.

ATP production rates were calculated based on the percent conversion of 2DOG to 2DOGP, the initial 2DOG concentration, incubation volume, and incubation time. In order to simultaneously assess H_2_O_2_ production, mitochondrial incubations were carried out in the presence of 10-acetyl-3,7-dihydroxyphenoxazine as described below.

### Quantification of ATP by NMR spectroscopy

NMR spectra were collected at 37°C on a Bruker Avance II 500 MHz NMR spectrometer. Mitochondrial samples were studied by acquiring two-dimensional (2D) ^1^H/^13^C HSQC NMR spectra using ^13^C-labeled 2DOG at C6-position ([6-^13^C]2DOG) as we recently described [12]. The amount of 2DOG and 2DOGP present in the NMR samples were quantitatively measured using the peak intensities of the assigned resonances of these compounds. NMR spectra were processed with the NMRPipe package [14] and analyzed using NMRView software [15].

### Metabolite quantification by NMR spectroscopy

Isolated mitochondria (0.1 mg/ml) were incubated at 37°C for 20 min in the presence of 10 mM uniformly ^13^C-labelled [^13^C_4_]-succinate, 5 mM 2DOG, and 5 U/ml HK with various ADP concentrations in a buffer containing 105 mM KCl, 10 mM NaCl, 5 mM Na_2_HPO_4_, 2 mM MgCl_2_, 10 mM HEPES, 1 mM EGTA, and 0.2% defatted BSA (pH 7.2). Immediately after incubation, the content was centrifuged to obtain the mitochondrial pellet (for metabolite studies within mitochondria) and supernatant (for metabolite studies external to mitochondria). These pellet and supernatant fractions were then extracted with 4% perchloric acid and centrifuged at 50,000 x g for 20 min at 4°C. Immediately after neutralization of the supernatants using 1 M KOH and centrifugation at 13,000 x g for 15 min 4°C to remove the precipitated salt, the supernatant was frozen and stored at -80°C for NMR studies. For NMR, 350 μl of the stored supernatant was added to 150 μl of 50 mM sodium phosphate, pH 7.4 in D_2_O for metabolite measurement. ^13^C and ^1^H assignments of malate, fumarate, and oxaloacetate (OAA) were obtained by using standard compounds. OAA was found to be unstable with a half-life (t_1/2_) about 14 hours when tested at pH 7.2 and temperature at 25°C. Therefore, soon after mitochondrial incubation, perchloric acid extraction was carried out as quickly as possible to destroy the mitochondrial enzymes and minimize the degradation of OAA. In addition, for precise metabolite quantification, known amounts of standard compounds were also subjected to parallel incubation and perchloric acid extraction. Both ^13^C/^1^H HSQC and HMQC spectra were collected at 25°C using a Bruker Avance II 800 MHz NMR spectrometer equipped with a sensitive cryoprobe for the perchloric acid-extracted samples for quantification of metabolites within mitochondria or metabolites released to the medium (external to mitochondria). All NMR spectra were processed using NMRPipe package [14] and analysed using NMRView [15]. Peak heights were used for quantification.

### Mitochondrial H_2_O_2_ production

H_2_O_2_ production was assessed simultaneously with ATP production as we have described [10] using the fluorescent probe 10-acetyl-3,7-dihydroxyphenoxazine (DHPA or Amplex Red, Life Technologies), a highly sensitive and stable substrate for horseradish peroxidase and a well-established probe for isolated mitochondria [16]. Fluorescence was measured and quantification carried out as we previously described [17]. Addition of catalase, 500 units/ml, reduced fluorescence to below the detectable limit, indicating specificity for H_2_O_2_. Addition of substrates to respiratory buffer without mitochondria did not affect fluorescence. DHPA does not interfere with ATP production or with NMR detection of 2DOGP [12].

### Opening of the MTP

MTP function was assessed as mitochondrial calcium retention capacity. Isolated mitochondria were exposed to repeated pulses of Ca^2+^ while monitoring extramitochondrial calcium fluorescence using Calcium Green ^™^-5N (Thermo Fisher Scientific) as the fluorescent probe. Mitochondria (0.15 mg/ml) were incubated at 37°C in 60 μl of respiration medium lacking EGTA but containing 120 mM KCl, 5 mM NaH_2_PO_4_, 2 mM MgCl_2_, 3 mM HEPES (pH 7.2), 5 units/ml hexokinase, 5 mM 2-deoxyglucose, 5 mM succinate, 0.3% fatty acid free BSA, and 1 μM Calcium Green^™^-5N. Calcium was added in sequential 5 μl pulses of 455 μM CaCl_2_. Pulse solutions contained 5 mM succinate to avoid dilution by the increase in volume due to repeated addition of calcium pulses. Fluorescence was monitored in a Fluostar Optima microplate reader (BMG Labtech). Measurements were taken every 16 seconds using an excitation wavelength of 485 nm and emission wavelength of 520 nm.

### NADH fluorescence

Intrinsic NADH fluorescence was monitored in isolated mitochondria as a marker of the mitochondrial NADH redox state. Isolated mitochondria were added to individual wells of 96-well black round-bottom microplates at a final concentrations of 0.1 mg/ml in 60 μl of respiration medium containing 105 mM KCl, 10 mM NaCl, 5 mM Na_2_HPO_4_, 2 mM MgCl_2_, 10 mM HEPES (pH 7.2), 1 mM EGTA, 0.2% defatted BSA, 5 units/ml hexokinase, and 5 mM 2-deoxyglucose. Respiratory substrates and ADP were present as indicated in the figure legends. Fluorescence was monitored in a Fluostar Optima microplate reader (BMG Labtech) at 37°C. Measurements were taken every 60 seconds using excitation and emission wavelengths of 355 nm and 450 nm, respectively.

### Statistics

Data were analyzed as indicated in the figure legends using GraphPad Prism (GraphPad Software, Inc., La Jolla, CA) or SigmaStat (Systat Software Inc., San Jose, CA). Significance was considered at p < 0.05.

## Results

### Mitochondrial function energized at complex II

**Fig 2:**
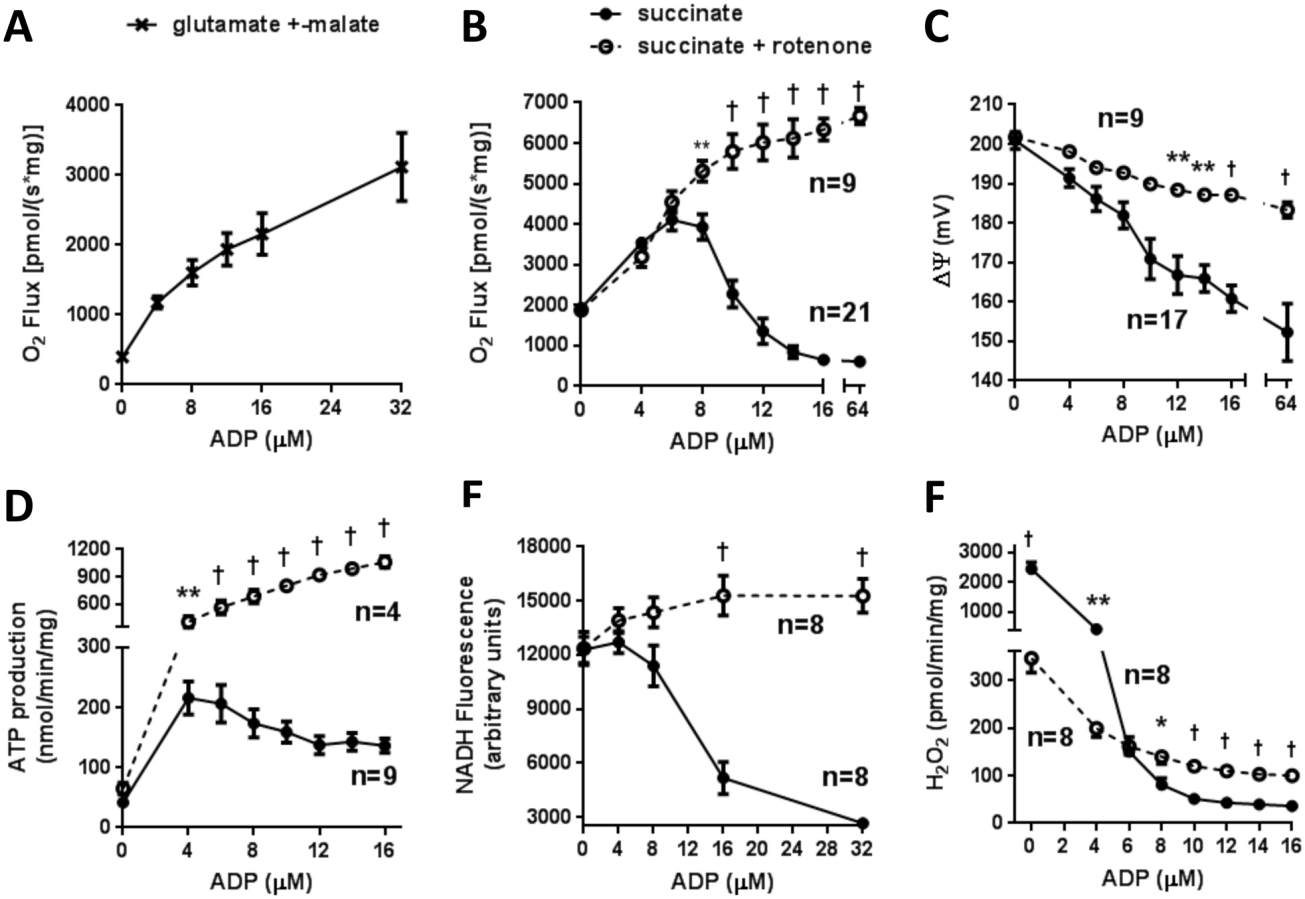
Mitochondrial functional parameters as a function of clamped [ADP] (mean ± SEM). ADP was added in incremental amounts in the presence of hexokinase and excess 2-deoxyglucose in order to clamp [ADP] and ΔΨ. ADP additions were made at approximately 90 sec intervals and completed well before depletion of chamber oxygen tension. (A) O_2_ flux in hindlimb muscle mitochondria respiring on 5 mM glutamate plus 1 mM malate (n = 3). (B-F) Parameters assessed in a total of 33 preparations of hindlimb skeletal muscle mitochondria respiring on 5 mM succinate with (open circles) or without (closed circles) 5 μM rotenone (number of isolates studied for each parameter are indicated in panels). (B) O_2_ flux. (C) Plateau values of ΔΨ recorded simultaneously with O_2_ flux. (D) ATP production measured as 2-deoxyglucose phosphate accumulation in multiwell plates over 20 min. (E) NADH fluorescence. (F) H_2_O_2_ production. * p < 0.05, ** p < 0.01, † p < 0.001 by t-test corrected for multiple comparisons by the Holm-Sidak method compared to succinate alone.

Respiration energized by succinate is often measured in the presence of rotenone to inhibit complex I, thereby preventing reverse electron transport [18] from complex II to complex I. As expected, for respiration on the complex I substrates, glutamate plus malate (Fig 2A), or on succinate plus rotenone (Fig 2B), we observed a progressive increase in O_2_ flux with incremental additions of clamped ADP. However, for respiration on succinate without rotenone, we observed an unanticipated pattern. O_2_ flux initially increased with increments in clamped [ADP] in parallel to respiration on succinate plus rotenone, but only until [ADP] reached concentrations of 6 to 8 μM (Fig 2B). This was followed by markedly decreased respiration as [ADP] was further increased. When this experiment was done in the presence of oligomycin to block ATP synthase, ADP increments had no effect on respiration (data not shown), confirming dependence on ATP production rather than ADP *per se*.

Plateau values for mitochondrial membrane potential (ΔΨ) in succinate-energized mitochondria decreased continuously as [ADP] was increased (Fig 2C). Using the 2DOG/hexokinase clamp, we also measured ATP production as formation of 2DOG phosphate by NMR spectroscopy, a very sensitive and specific method that we recently described [10, 12]. ATP production by succinate-fueled mitochondria manifested a similar biphasic response to [ADP] as seen for respiration (Fig 2D). NADH fluorescence did not change at low [ADP] but, in the absence of rotenone, decreased after 4 μM ADP (Fig 2E). The production of reactive oxygen species (ROS) was very high under state 4 conditions in the absence of rotenone (Fig 2F), as expected based on previous data from our laboratory [17, 19] and others [18]. Also, as expected ROS dropped markedly with added ADP and reduction in ΔΨ.

### Lack of involvement of the mitochondrial permeability transition pore (MTP)

**Fig 3:**
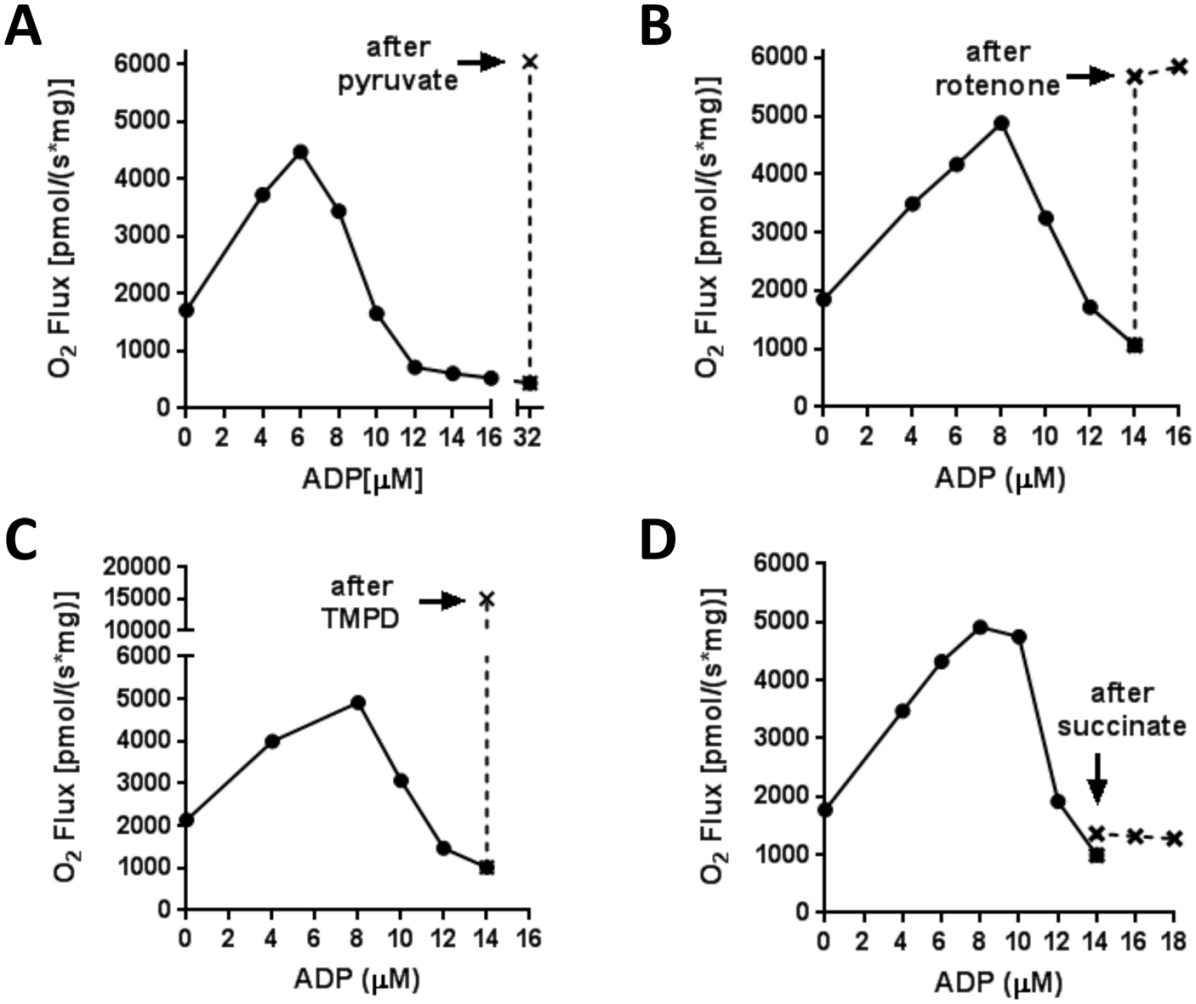
Mitochondria respiring on succinate are not irreversibly depolarized. O_2_ flux in mitochondria respiring as in Fig 2B on 5 mM succinate could be rescued by addition of 5 mM pyruvate (panel A), 5 μM rotenone (panel B), or 2 mM ascorbate + 0.5 mM N,N,N',N'-Tetramethyl-p-phenylenediamine dihydrochloride (TMPD) to provide electrons directly to complex IV (panel C); indicating that the functional status of the organelles was preserved. O_2_ flux could not be rescued by adding additional succinate to a final concentration of 10 mM (panel D). Solid lines indicate O_2_ flux prior to addition of the compound indicated. Dotted lines indicate O_2_ flux after addition. Each panel is representative of 2–3 repetitions.

We questioned whether the MTP might have a mechanistic role underlying the biphasic dependence of O_2_ flux on the concentration of added ADP. Lowering membrane potential is known to favor opening of the MTP [20, 21]. Moreover, there is some evidence that OAA may contribute to pore function [22]. Complete MTP opening, destructive to mitochondrial function, did not occur under the conditions of our studies, since the loss of respiration at high [ADP] could be completely and rapidly rescued by addition of rotenone, pyruvate, or by electron donation directly to complex IV by ascorbate + 0.5 mM N,N,N',N'-tetramethyl-p-phenylenediamine dihydrochloride (TMPD), but not by additional succinate (Fig 3). On the other hand, transient opening or “flickering” of the MTP can occur [23]. Therefore, we examined the relationship of O_2_ flux to [ADP] in mice deficient in CypD, a critical component of the MTP [23, 24]. We found no difference in succinate-supported respiration by mitochondria isolated from mice deficient in CypD compared to controls (S1A Fig) while we were able to document the effectiveness of the KO modification (S1B and S1C Fig).

### Respiratory dependence on titrated membrane potential

**Fig 4:**
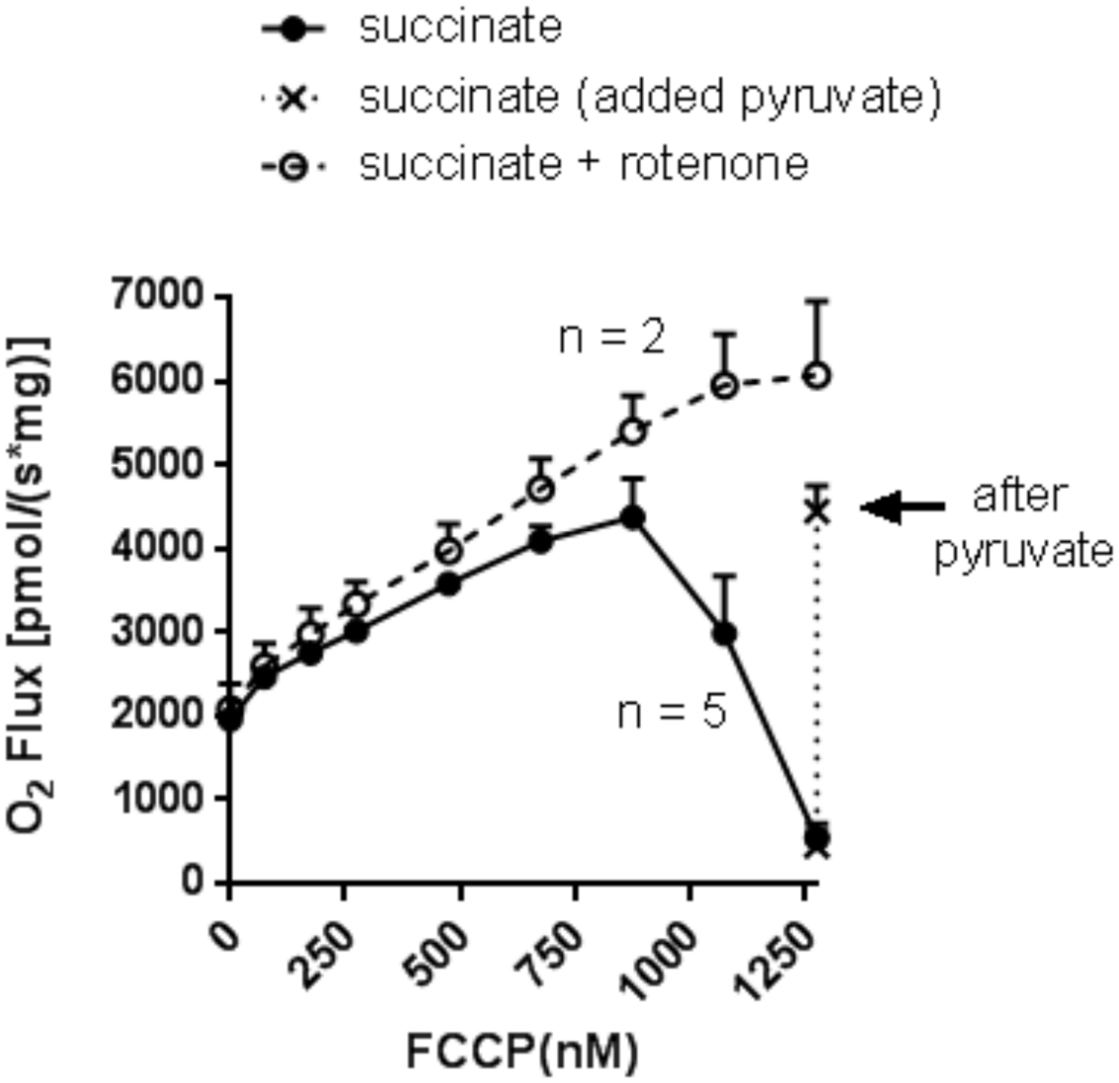
Succinate-energized mitochondrial respiration as a function of incremental FCCP additions to titrate membrane potential independent of added ADP. Incubations were carried out as in Fig 1B in the presence or absence of 5 μM rotenone. Solid line (no rotenone) indicates O_2_ flux prior to addition of 5 mM pyruvate. Dotted line (no rotenone) indicates O_2_ flux after addition of pyruvate. Dashed line indicates O_2_ flux in the presence of rotenone. Data represent (mean ± SEM) with the number or repetitions indicated.

To assess the effect of membrane potential *per se* (no added ADP) on succinate-energized O_2_ flux, we measured respiration while titrating with FCCP (Fig 4). We observed the same biphasic respiratory pattern. Moreover, as was the case for ADP titration, respiration could be rescued by pyruvate addition.

### Loss of O_2_ flux did not result from time of incubation or reduction in O_2_ tension

**Fig 5:**
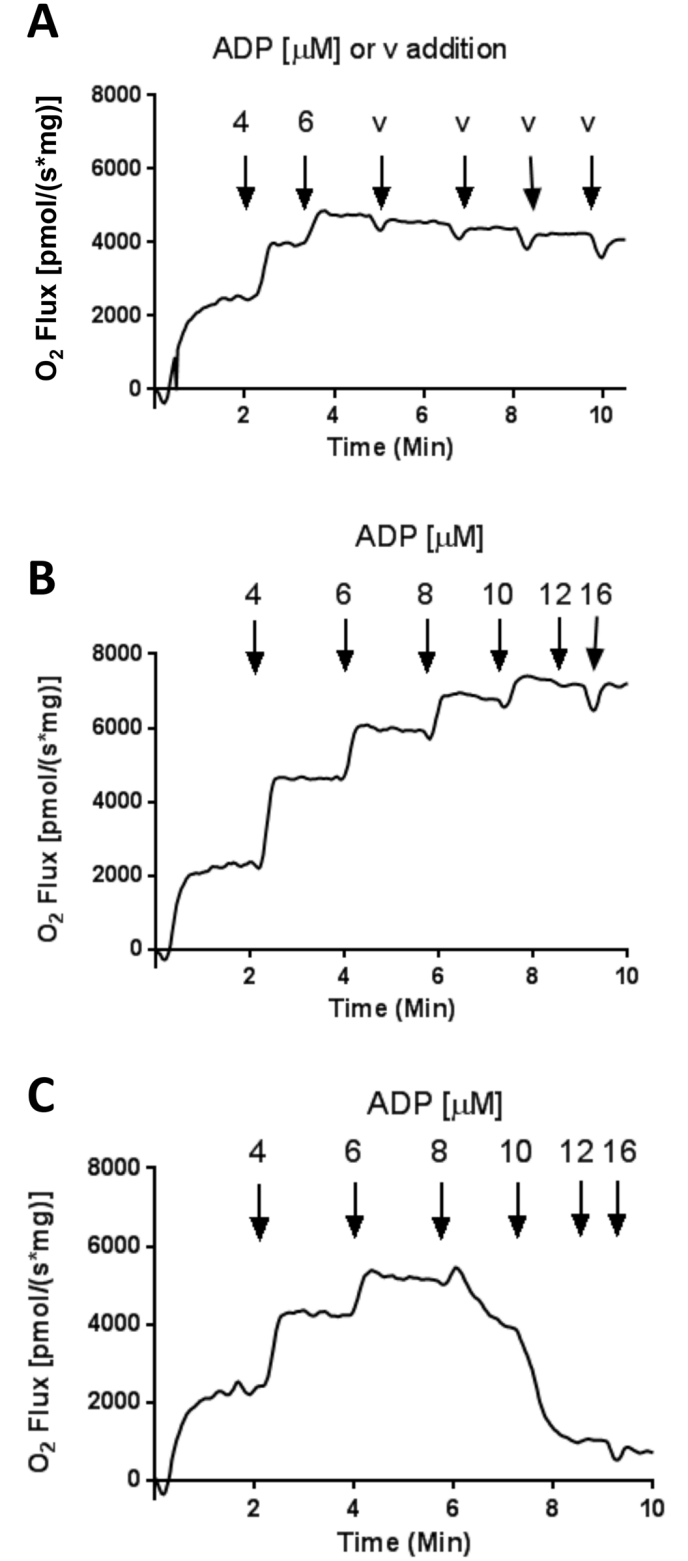
O_2_ flux in hindlimb muscle mitochondria respiring on succinate and treated with incremental additions of ADP or vehicle (water) in the presence of HK and 2DOG. (A) Mitochondria respiring on 5 mM succinate without rotenone. ADP was incremented to 6 μM and maintained at that concentration with vehicle (v, water) injections indicated by arrows to maintain volumes equivalent to the incubations of panels B and C. (B) Mitochondria respiring on 5 mM succinate plus 5 μM rotenone. ADP was incremented to achieve the indicated (arrows) concentrations. (C) Mitochondria respiring on 5 mM succinate without rotenone. ADP was incremented to achieve the indicated (arrows) concentrations. These traces are representative of 3 repeated experiments.

In measuring O_2_ flux and ΔΨ, we sequentially added incremental amounts of ADP (sequential clamped respiratory states) to mitochondria within the Oxygraph respiratory chamber over a period of about 10 minutes. The observed dramatic fall in respiration after 6–8 μM ADP for succinate (alone)-energized mitochondria was clearly not due to incubation time. This is clear since addition of ADP up to 6 μM followed by maintenance of that concentration resulted in maintenance of respiration and ΔΨ over time (Fig 5A), whereas O_2_ flux increased in stepwise fashion with increasing ADP when mitochondria were fueled with 5 mM succinate plus 5 μM rotenone (Fig 5B), and O_2_ flux increased then decreased in stepwise fashion with increasing ADP for mitochondria fueled with 5 mM succinate alone (Fig 5C). Mitochondrial respiration will clearly cease if O_2_ tension drops over time. However, in our experiments, O_2_ tension did not drop to < 40% of atmospheric and mitochondrial respiration is known to be robust until much lower O_2_ tension is reached. Moreover, if O_2_ tension had dropped to such a critical level, we would not have seen the marked increases induced by pyruvate, rotenone, or TMPD (Figs 3 and 4).

We point out that incubation time was likewise not a factor with respect to our measurements of ATP production, NADH, and generation of ROS. As opposed to O_2_ flux and ΔΨ, these parameters were determined under constant clamped ADP throughout 20 min incubation times (see methods).

### Respiration at different clamped concentrations of ADP unchanged over time (no titration)

**Fig 6:**
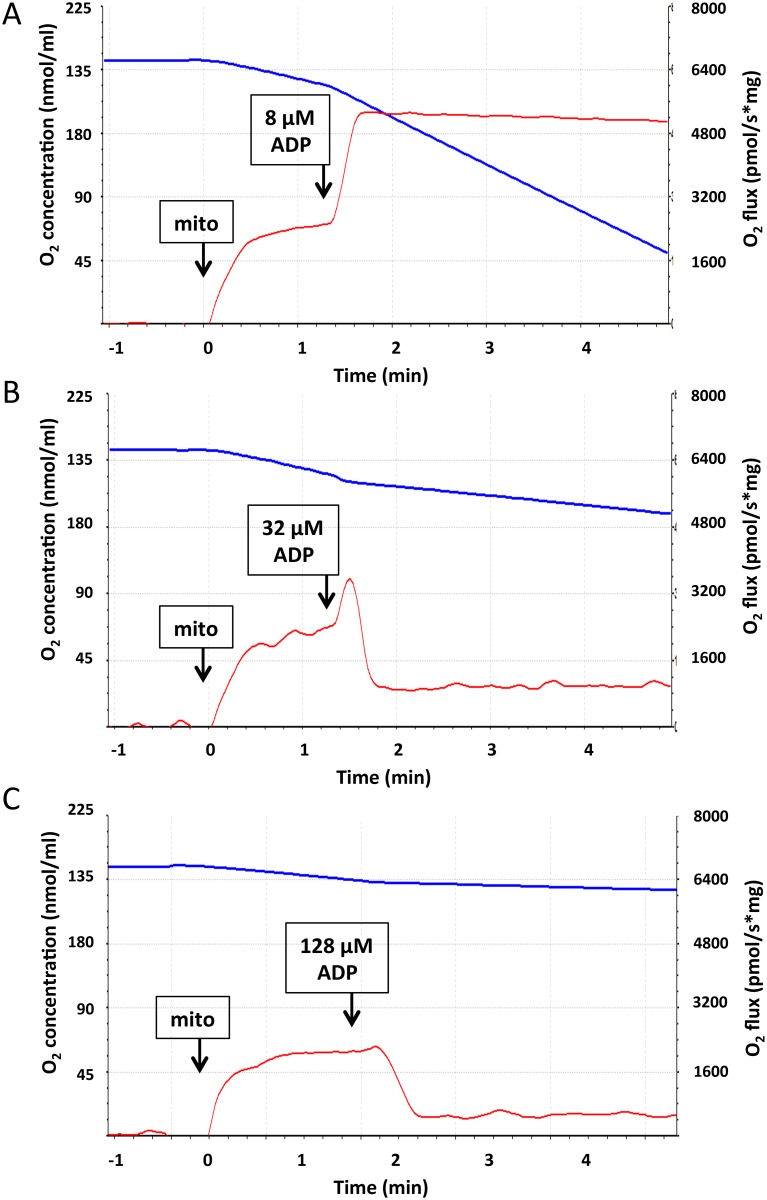
O_2_ flux in in mitochondria energized with 5 mM succinate in the absence of rotenone as affected by ADP clamped at the concentrations indicated. In these experiments ADP was maintained, rather than titrated, at the same concentration throughout the incubation period. A) [ADP] = 8 μM. B) [ADP] = 32 μM. C) [ADP] = 128 μM. Mitochondria (mito), 0.05 mg/ml, and ADP were added at the times indicated (arrows). O_2_ concentration (blue) and O_2_ flux (red) are depicted graphically. X-axis depicts time relative to the addition of mitochondria (time 0). These experiments are representative of three replicates. Data show actual Oxygraph tracings with axis labels added for clarity.

As shown in Fig 6A, addition of ADP at 8 μM induced a smooth rise and plateau in O_2_ flux. However, addition of 32 μM ADP caused an initial transient rise in O_2_ flux which lasted only several seconds followed by a fast and substantial drop to levels below that seen in the absence of ADP (Fig 6B). ADP at 128 μM caused only an initial small rise in O_2_ flux followed by a fast and large drop (Fig 6C).

### Metabolite data

**Fig 7:**
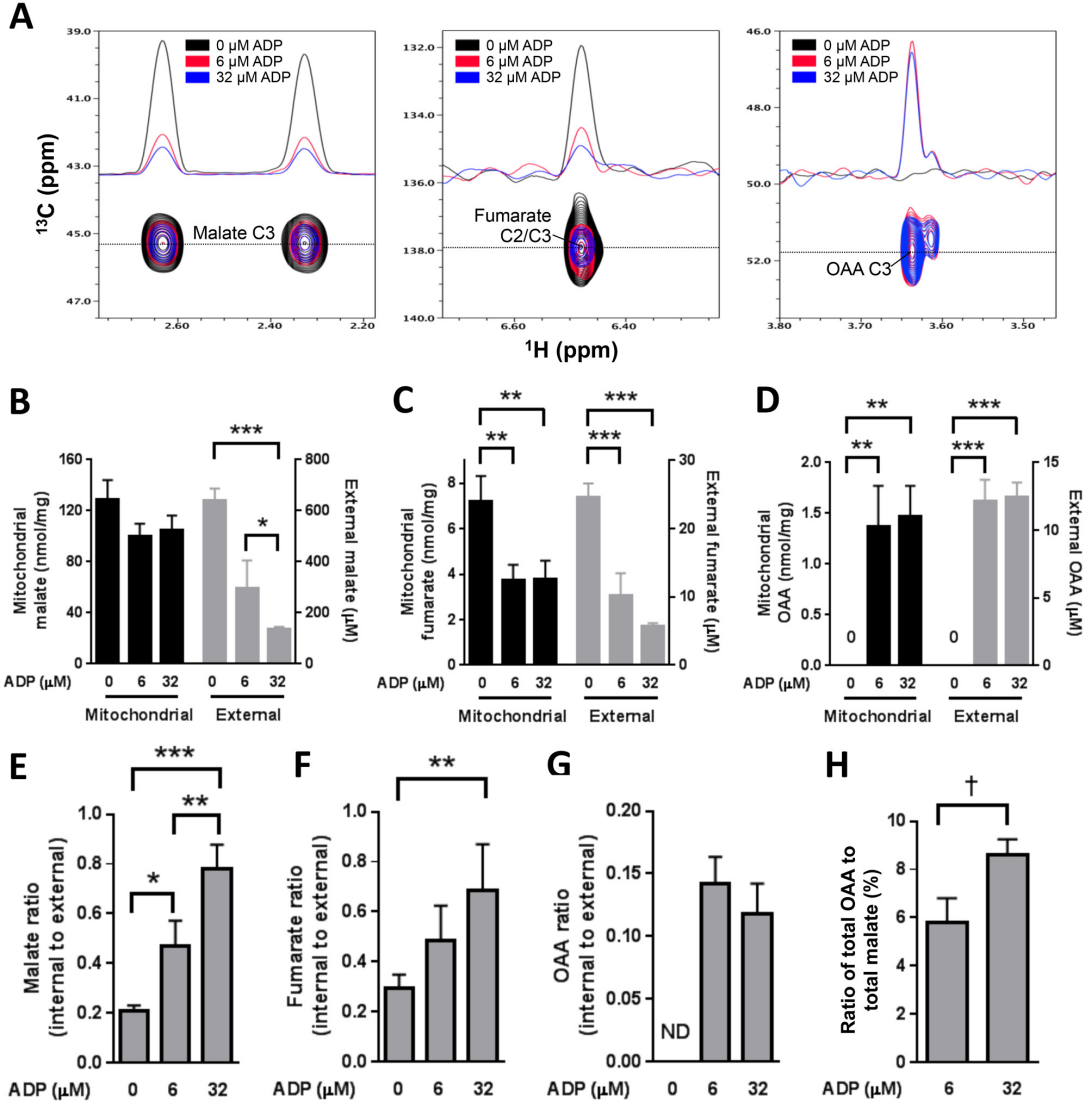
Metabolite accumulation determined by NMR spectroscopy in mitochondria incubated for 20 min in the absence (0 ADP) or presence of low (6 μM) or high (32 μM) ADP. Mitochondria were energized by 10 mM uniformly labeled [^13^C]succinate in the presence of HK and 2DOG. (A) Representative 2D ^13^C/^1^H HMQC spectra for malate and fumarate and HSQC spectra for oxaloacetate (OAA) of the medium after mitochondrial incubation in the presence of various ADP concentrations as indicated by spectral color. One-dimensional slices through the cross-peaks as indicated by the dotted line are also shown. (B-D) Content of indicated metabolite: malate, fumarate, or OAA within mitochondria (nmol/mg mitochondrial protein) or external (μM in medium) at end of incubation. (E-G) Metabolite ratios of internal mitochondrial to external medium from data of panels B-D. The ratios are expressed as mitochondrial content (nmol/mg) to external concentration since it is difficult to be sure of mitochondrial volumes. ND, not determined. Data represent mean ± SEM, n = 6. * p < 0.05, ** p < 0.01, or *** p < 0.001 by repeated measures ANOVA and multiple comparisons by the Holm-Sidak method or rank test for non-parametric data. (H) Ratio of total (internal plus external) OAA to total malate (mean ± SEM, n = 6, † p = 0.001 by 2 tailed, paired t-test).

Metabolites were measured by NMR spectroscopy (Fig 7A) within and external to mitochondria when incubated with uniformly ^13^C-labeled succinate. Measurement of OAA was not trivial because, unlike malate and fumarate, OAA is unstable [25] requiring rigid assessment of stability over time, appropriate controls for metabolite deterioration, and rapid sample processing (see methods). The metabolite measurements represent the amounts accumulated within and external to the mitochondria over 20 minute incubation times. External molar amounts were greater than internal, but because of the small internal volume of the mitochondria (estimated at roughly 1 μl/mg mitochondrial protein), mitochondrial concentrations were in the range of 100 to 1000 fold greater than external (depending on conditions and metabolite).

We detected a marked decrease in malate and fumarate in the incubation medium (Fig 7B and 7C) and a significant drop in fumarate inside mitochondria (Fig 7C) at 6 and 32 μM ADP relative to 0 μM. At 0 μM ADP, when NADH is high, OAA was undetectable, but clearly present at similar concentrations at 6 and 32 μM ADP both within and external to mitochondria (Fig 7D). Malate and fumarate exit from mitochondria were significantly reduced with increasing ADP as evidenced by the increasing internal to external mitochondrial ratios (Fig 7E and 7F), while OAA exit from mitochondria did not change (Fig 7G). The ratio of total OAA over total malate in both internal and external mitochondria increased with increasing ADP (Fig 7H). As expected, citrate could not be detected (data not shown). When mitochondria were incubated at 0, low, or high [ADP] in the presence of rotenone, OAA was undetectable while malate and fumarate increased with increasing [ADP] (data not shown, n = 3 for each [ADP]).

### Endogenous malate inhibits respiration by succinate-energized mitochondria

**Fig 8:**
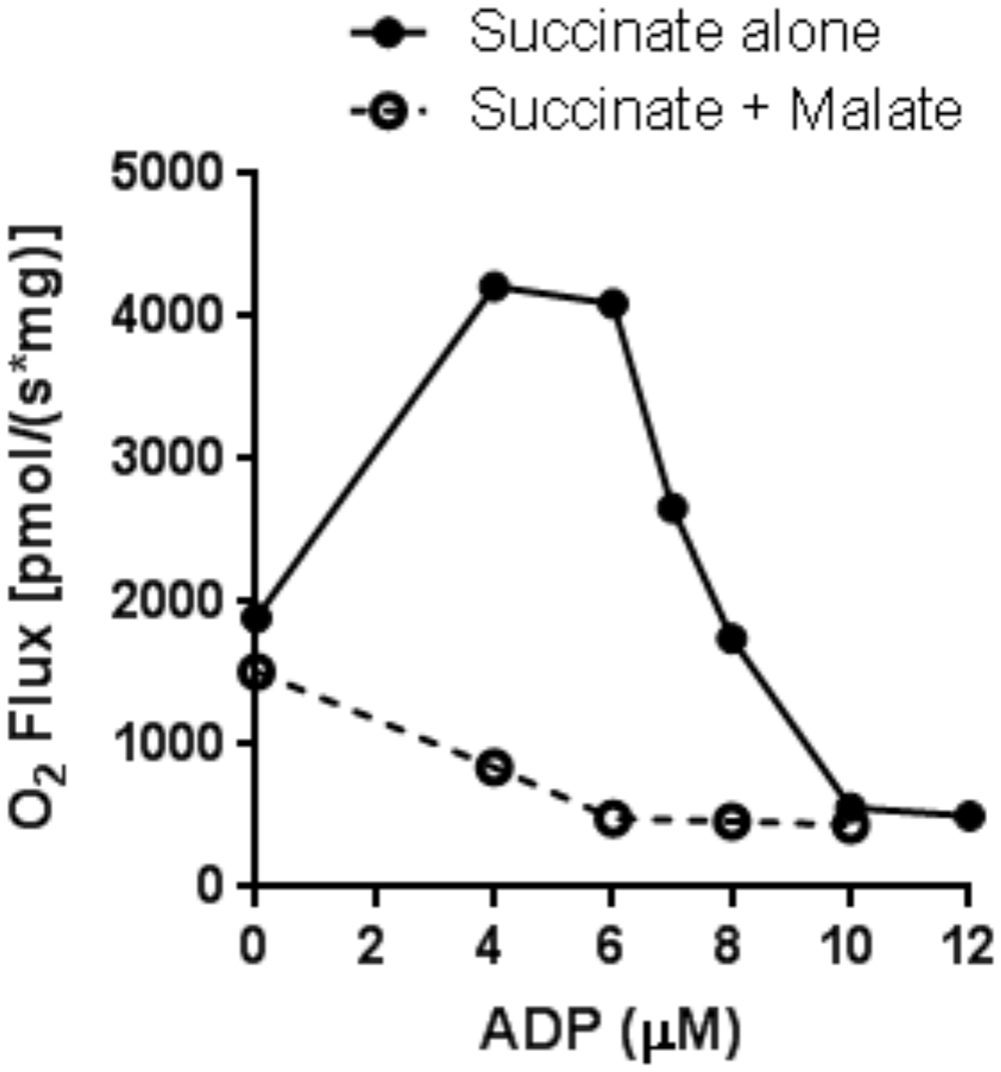
O_2_ flux versus [ADP] in mitochondria energized with 5 mM succinate alone or 5 mM succinate plus 1 mM malate. Mitochondria were incubated as described in the legend to Fig 2 in the presence of HK and 2DOG. Data are representative of triplicate experiments.

If malate exit were important in regulating metabolite flux to OAA, a potent inhibitor of succinate dehydrogenase (SDH) [26, 27], we would expect endogenous addition of malate to reduce respiration. As shown in Fig 8, this was clearly the case.

### Succinate-energized respiration in mitochondria of other tissues as a function of [ADP]

**Fig 9:**
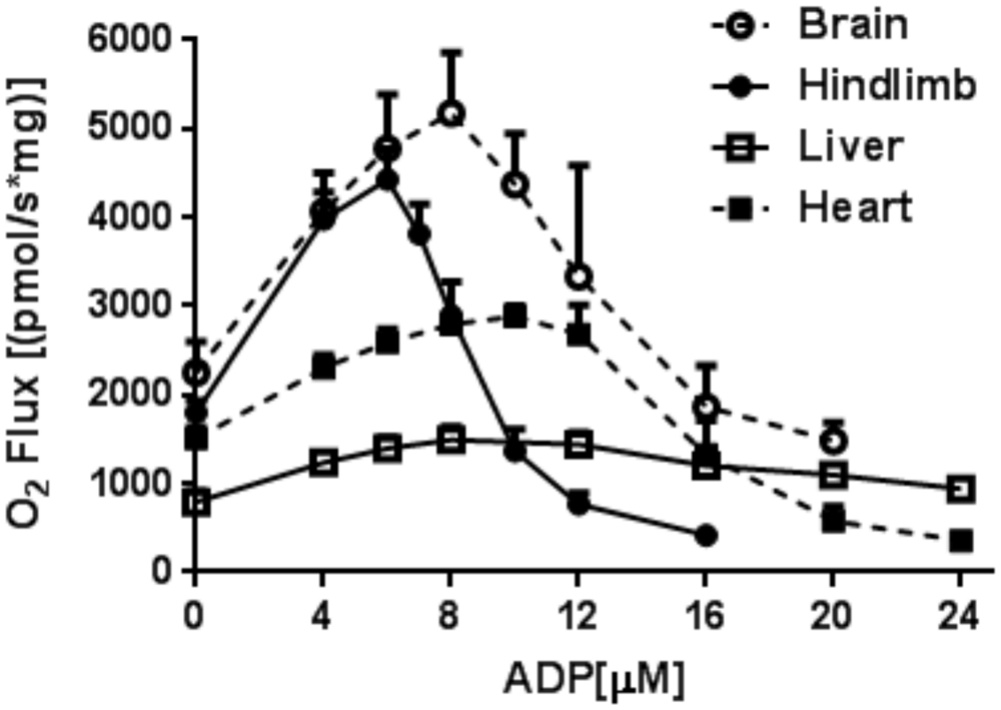
O_2_ flux in mitochondria isolated from different tissues as a function of clamped [ADP]. Mitochondria were energized by 5 mM succinate and incubated as described in the legend to Fig 2 in the presence of HK and 2DOG (data represent mean ± SE, n = 3).

We also examined O_2_ flux in succinate-fueled mitochondria isolated from mouse brain, heart, and liver. Again, we observed a biphasic relationship of respiration to [ADP] in heart and brain mitochondria and to a lesser extent in liver mitochondria (Fig 9).

## Discussion

The major new finding reported here is the biphasic pattern for respiration on the complex II substrate succinate. This up and down pattern (Fig 2B) is unique in that O_2_ utilization on different energy substrates has been considered to steadily increase with decreasing ΔΨ by uncoupling or as one titrates with ADP (also decreasing ΔΨ).

Note that we do not claim that the decrease in respiration at higher ADP is related to the respiratory state moving closer to state 3. Rather we show that both respiration and ATP production (which determines the respiratory state) decrease beyond a certain level of ADP-induced reduction in ΔΨ. Thus, our findings do not negate the well-established proportionality of O_2_ flux to respiratory state.

Studies dating back over 50 years describe inhibition of succinate oxidation by chemical uncoupling associated with high concentrations of OAA and reversible by pyruvate or other energy substrates [28–31]. Moreover, tissue specific OAA inhibition of complex II, which has conceptual overlap with the voltage-dependent regulation described herein, in mitochondria respiring on succinate has been described [32–34]. This has been termed “intrinsic inhibition” and felt to offer protection from excess substrate flux and associated ROS production by reverse transport form complex II to complex I [32]. However, we could find no report of the biphasic [ADP] dependence as reported here. We believe this is because most studies of isolated mitochondria have not been carried out under titration of the respiratory state or of membrane potential and because studies that did involve ADP regeneration [2–8] did not rigidly clamp ΔΨ and were mostly carried out using liver mitochondria wherein the biphasic nature of O_2_ flux on succinate was much less evident (Fig 9).

Since we found no prior reports of this respiratory pattern, we carried out several studies to address the underlying mechanism. Our results show that irreversible opening (Fig 3A, 3B and 3C) or flickering of the MTP (S1 Fig) cannot be implemented. ROS, in the form of superoxide, is well known to be generated via succinate-driven reverse electron transport (RET) and is converted to H_2_O_2_ by matrix superoxide dismutase [17, 18]. However, ROS do not explain the biphasic change in O_2_ flux (Fig 2B) since, unlike O_2_ flux, H_2_O_2_ production steadily decreases with added ADP (Fig 2F). It is of incidental note that beyond 8 μM ADP, ROS in the absence of rotenone dropped even lower than that observed when rotenone was present, presumably because of the marked drop in respiration.

**Fig 10:**
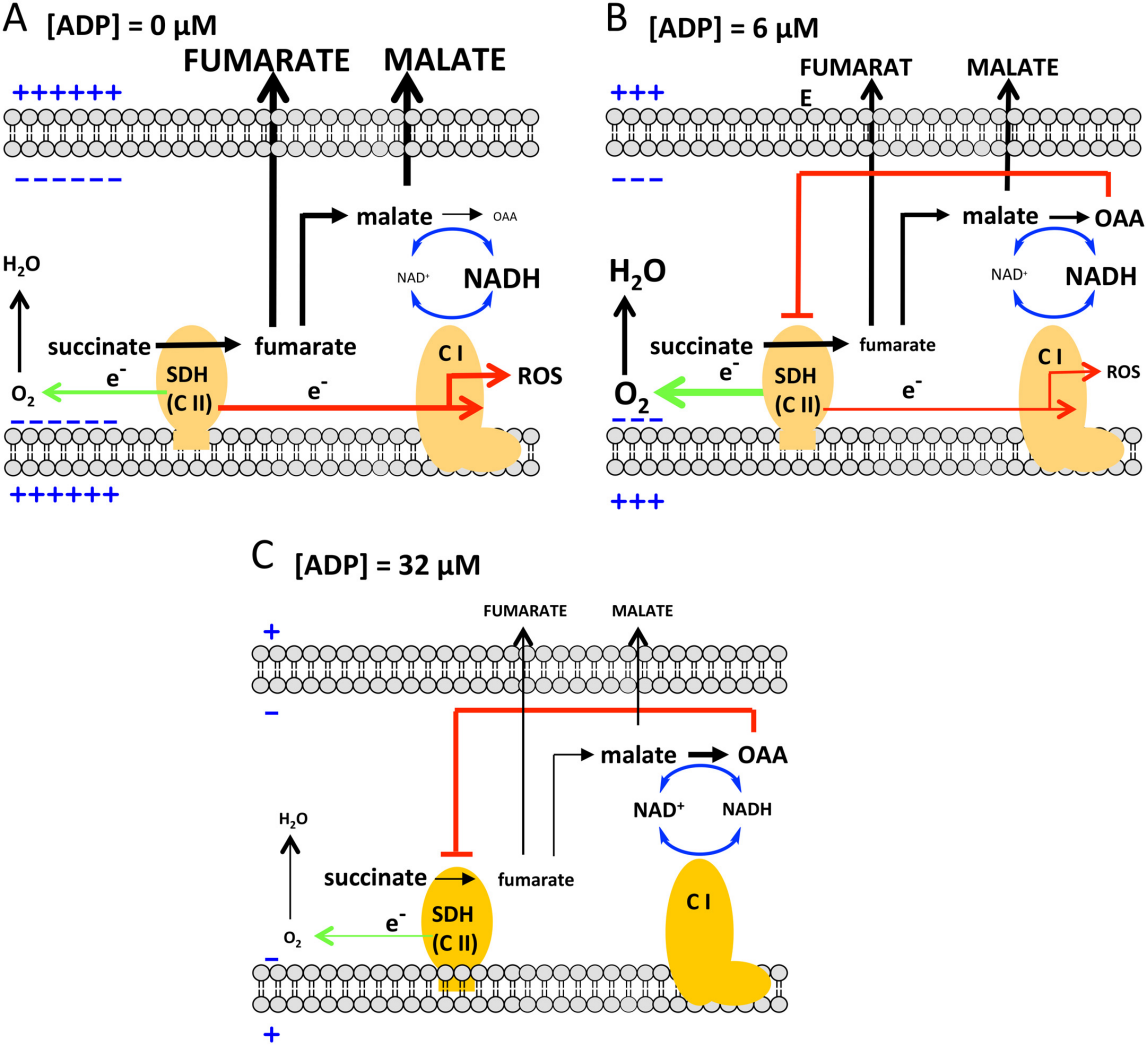
Mechanistic regulation of respiration by succinate-fueled mitochondria at differing clamped ADP levels. (A) [ADP] = 0 μM, (B) [ADP] = 6 μM, (C) [ADP] = 32 μM. O_2_ flux is proportional to forward electron transport (green arrows) leading to downstream O_2_ consumption. O_2_ flux is highest at [ADP] = 6 μM, lowest at [ADP] = 32 μM, and intermediate at [ADP] = 0 μM in accord with the data of Fig 2B. The extent of O_2_ flux is regulated by the net effects of membrane potential (blue charge symbols), oxaloacetate (OAA) inhibition of succinate dehydrogenase (SDH), the extent of reverse electron transport (red arrows), the extent of reduction of the NADH/NAD^+^ redox couple, and metabolite exit. The changes in these factors are supported by our metabolite and functional data as detailed in the text. Thickness of arrows or lines and size of text depict magnitude of effects or concentrations. C I and C II depict complexes 1 and 2. Note that OAA cannot undergo further metabolism in the absence of a source of acetyl CoA (and evidenced in that we were unable to detect ^13^C-citrate).

Our metabolite data suggest that the mechanism underlying the biphasic response of succinate-energized respiration involves the interplay of multiple basic metabolic factors. We propose the following. Initially, in the absence of added ADP (Fig 10A), RET from complex II to complex I is high; a well-known characteristic of state 4 respiration [18] and consistent with the observed substantial ROS production (Fig 2F). As a result, NADH is maintained in the reduced state; as both observed (Fig 2E) and expected [18]. This encourages malate exit and prevents malate conversion to OAA, both factors favoring respiration. However, respiration is moderate in this circumstance (Fig 2B), limited by high membrane potential.

With added ADP in low amounts (Fig 10B), the resultant decrease in potential is enough to drive respiration. That the initial event is decreased potential is supported in that the response to [ADP] titration is mimicked by stepwise chemical uncoupling (Fig 4). RET, which is highly sensitive to ΔΨ [18], is likewise reduced; as evidenced by the reduction in ROS (Fig 2F), a marker of reverse transport from complex II [18, 35]. The decrease in RET favors NADH oxidation although we observed only a slight decrease in NADH fluorescence (Fig 2E). However, some NADH/NAD^+^ cycling must occur as malate is driven to OAA. Malate still exits mitochondria, albeit in lower amounts. The net effect is increased respiration.

As ADP is increased to higher concentrations (Fig 10C), potential is further reduced. This further reduces RET, which is then much less effective in maintaining NADH reduction; thereby favoring OAA production and OAA-inhibition of SDH. However, we do not propose that OAA inhibition is the only factor limiting succinate energized respiration. Note that malate and fumarate exit at 32 μM ADP are further reduced (Fig 7B, 7C, 7E and 7F), suggesting that the mitochondrial shuttles for these metabolites are potential or voltage-dependent. This reduction in malate and fumarate exit will (in addition to OAA feedback) limit respiration due to backpressure on SDH. We point out that OAA feedback also cannot explain the decrease in respiration below the level at state 4 (Fig 2B), since that would represent overcorrection.

To summarize the above, our data suggest that biphasic ADP-dependent respiration triggered by electron donation at complex II is regulated by multiple factors. These include lowering of ΔΨ with subsequent coordinate changes in directional electron flow, NADH oxidation, OAA accumulation, and metabolite exit.

The mechanistic events as depicted in Fig 10 raise certain additional questions. Note that mitochondrial OAA and malate concentrations are similar at 6 and 32 μM ADP (Fig 7B and 7D), so we would expect the extent of OAA inhibition of SDH-driven respiration to be similar. Moreover, NADH oxidation, based on fluorescence at low [ADP] (Fig 2E), is only slightly less than at 0 [ADP]. So, given that NAD^+^ is needed for the malate dehydrogenase reaction, we need to explain how the OAA concentration at 6 μM ADP is roughly the same as at 32 μM ADP.

With regard to NAD^+^ needed for malate conversion to OAA, the K_eq_ for malate dehydrogenase specifies a reaction far to the left. Indeed, this is consistent with the much greater malate than OAA concentrations that we observed. But only a small amount of OAA may be needed to inhibit SDH. So, at low [ADP], only small changes in the NADH/NAD^+^ redox state may suffice for OAA inhibition of SDH. Although larger amounts of OAA might be expected at higher [NAD^+^], this is limited since OAA inhibition of SDH also inhibits its own production. It might also be possible (although clearly speculative) that the malate dehydrogenase K_cat_ (regulating malate turnover to OAA) is limiting rather than the amounts of available NAD^+^. If so, then the differences in extent of NADH oxidation at 6 versus 32 μM ADP may not be critical.

Why is respiration inhibited at high [ADP] even though OAA concentrations are similar at 6 and 32 μM ADP? This may occur since the pathways (Fig 10) involve metabolite exit as well as substrate conversions. Although mitochondrial concentrations of fumarate and malate are similar at 6 and 32 μM ADP, reduced exit of these metabolites at 32 μM ADP in the face of a malate dehydrogenase K_eq_ favoring malate would create back pressure on SDH. We also point out that the similar OAA concentrations at 6 and 32 μM ADP, even though accurate, may be somewhat misleading if not considered carefully. This is because we examined metabolite accumulation over 20 min, while Fig 6 shows that at 32 μM ADP there is a rise followed by a drastic drop in respiration within the first minute after adding ADP. So it is possible that much of the OAA generated at 32 μM ADP occurred early in the 20 minute period or, in other words, may have been transiently higher than at 6 μM ADP.

The importance of the NADH/NAD^+^ redox couple is evident in the functional data in the presence of rotenone (Fig 2B–2F). Rotenone is well known to inhibit complex I and prevent NADH oxidation, enabling vigorous respiration unlimited by the above consequences of oxidation to NAD^+^. This is consistent with our observation that OAA was not detectable in mitochondria incubated in the presence of rotenone.

Our findings are also important in relation to current dogma asserting that to assess O_2_ flux on succinate in robust fashion, respiration should be measured in the presence of rotenone to inhibit complex I. However, our data suggest that robust O_2_ flux on succinate can be measured without rotenone at least up to a certain intermediate respiratory state.

A limitation is that we carried out detailed studies only in skeletal muscle mitochondria. Nonetheless, we did examine O_2_ flux in succinate-fueled mitochondria isolated from mouse brain, heart, and liver. As in skeletal muscle, we observed a similar biphasic relationship of O_2_ flux to [ADP] in heart and brain mitochondria, albeit with slightly different quantitative dynamics (Fig 9). For liver mitochondria the biphasic nature was evident but not as well defined (Fig 9).

An unanswered issue regards the exact process or processes controlling metabolite exit. A very small part could be due to damaged mitochondria since no preparation can be considered 100% intact. However, we did purify our mitochondria and document about 96% intact organelles (see methods). And clearly, for malate and fumarate, the five-fold differences in external metabolite amounts between incubations at 0 and 32 μM ADP (Fig 7B and 7C) indicate much more than metabolite leak through damage.

We cannot rule out other factors mechanistically involved in the biphasic dependence of succinate-energized respiration on [ADP]. For example, one might speculate regarding conformational change in CI [36], sulfhydryl modifications, acetylation regulated by NAD [37], or other factors. However, this does not negate or detract from the metabolic interactions that we report here. Further, we contend that it should not be surprising that processes as basic as mitochondrial oxygen consumption might be regulated in complex and interdependent ways.

In summary, we describe a previously unrecognized voltage-dependent regulation of mitochondrial function at complex II. We propose that the primary mechanistic factor is ADP-induced reduction in ΔΨ resulting in coordinate changes including altered directional electron flow, altered NADH/NAD^+^ redox cycling, reduced metabolite exit, and OAA inhibition of SDH. Moreover, this work suggests that mitochondrial function might best be studied using an approach that allows measurements at intermediate and more physiologic respiratory conditions as opposed to studies only directed at state 4 or state 3 respiration.

## Supporting Information

S1 Fig Mitochondria respiring on succinate are not affected by the mitochondrial permeability transition pore. (A) O_2_ flux determined in hindlimb muscle mitochondria of control (C) and CypD knock out (KO) mice respiring on succinate (5 mM) in the presence or absence of rotenone (rot) (5 μM) incubated as in the legend to Fig 2 (main text) in the presence of HK and 2DOG. Data represent mean ± SE, n = 4 per group. No significant differences were observed between C and KO mice. (B) Calcium green fluorescence by mitochondria from a C and KO mouse. Mitochondria were energized by 5 mM succinate alone in the absence of ADP (state 4) with repeated administration of 5 μl of calcium chloride (455 μM) in 60 μl incubation volumes. (C) Incubations as in panel B, but in the presence of rotenone (5 μM). Panels B and C are representative of experiments repeated for all four C and KO mice using mitochondria from the preparations utilized for the experiments in panel A. Data show delayed opening of the MTP in KO mitochondria as manifest by greater Ca^2+^ retention, thus confirming the effectiveness of the KO perturbation. The difference in Ca^2+^ retention time between panels B and C (greater with rotenone) is an incidental finding but is compatible with the much greater ROS production at 0 ADP in the absence versus presence of rotenone (Fig 2F), even though potential did not differ at 0 ADP (Fig 2C).
(PDF)
