# Supplementary material for: Voltage-Dependent Regulation of Complex II Energized Mitochondrial Oxygen Flux
Source: PLoS One. 2016 May 6;11(5):e0154982. doi: 10.1371/journal.pone.0154982 (PMC4859540; doi:10.1371/journal.pone.0154982)
Supplement: S1 Fig — (A) O2 flux determined in hindlimb muscle mitochondria of control (C) and CypD knock out (KO) mice respiring on succinate (5 mM) in the presence or absence of rotenone (rot) (5 μM) incubated as in the legend to Fig 2 (main text) in the presence of HK and 2DOG. Data represent mean ± SE, n = 4 per group. No significant differences were observed between C and KO mice. (B) Calcium green fluorescence by mitochondria from a C and KO mouse. Mitochondria were energized by 5 mM succinate alone in the absence of ADP (state 4) with repeated administration of 5 μl of calcium chloride (455 μM) in 60 μl incubation volumes. (C) Incubations as in panel B, but in the presence of rotenone (5 μM). Panels B and C are representative of experiments repeated for all four C and KO mice using mitochondria from the preparations utilized for the experiments in panel A. Data show delayed opening of the MTP in KO mitochondria as manifest by greater Ca2+ retention, thus confirming the effectiveness of the KO perturbation. The difference in Ca2+ retention time between panels B and C (greater with rotenone) is an incidental finding but is compatible with the much greater ROS production at 0 ADP in the absence versus presence of rotenone (Fig 2F), even though potential did not differ at 0 ADP (Fig 2C). (PDF) [file pone.0154982.s001.pdf]

Supplemental Data

Voltage-Dependent Regulation of Complex II Energized Mitochondrial Oxygen Flux

Fan Bai[1], Brian D. Fink[2], Liping Yu[1,3], and William I. Sivitz[2#]

[1] Department of Biochemistry, [2] Department of Internal Medicine / Endocrinology and Metabolism, [3]NMR Core Facility, University of Iowa and the Iowa City Veterans Affairs Medical Center, Iowa City, IA, 52242 USA

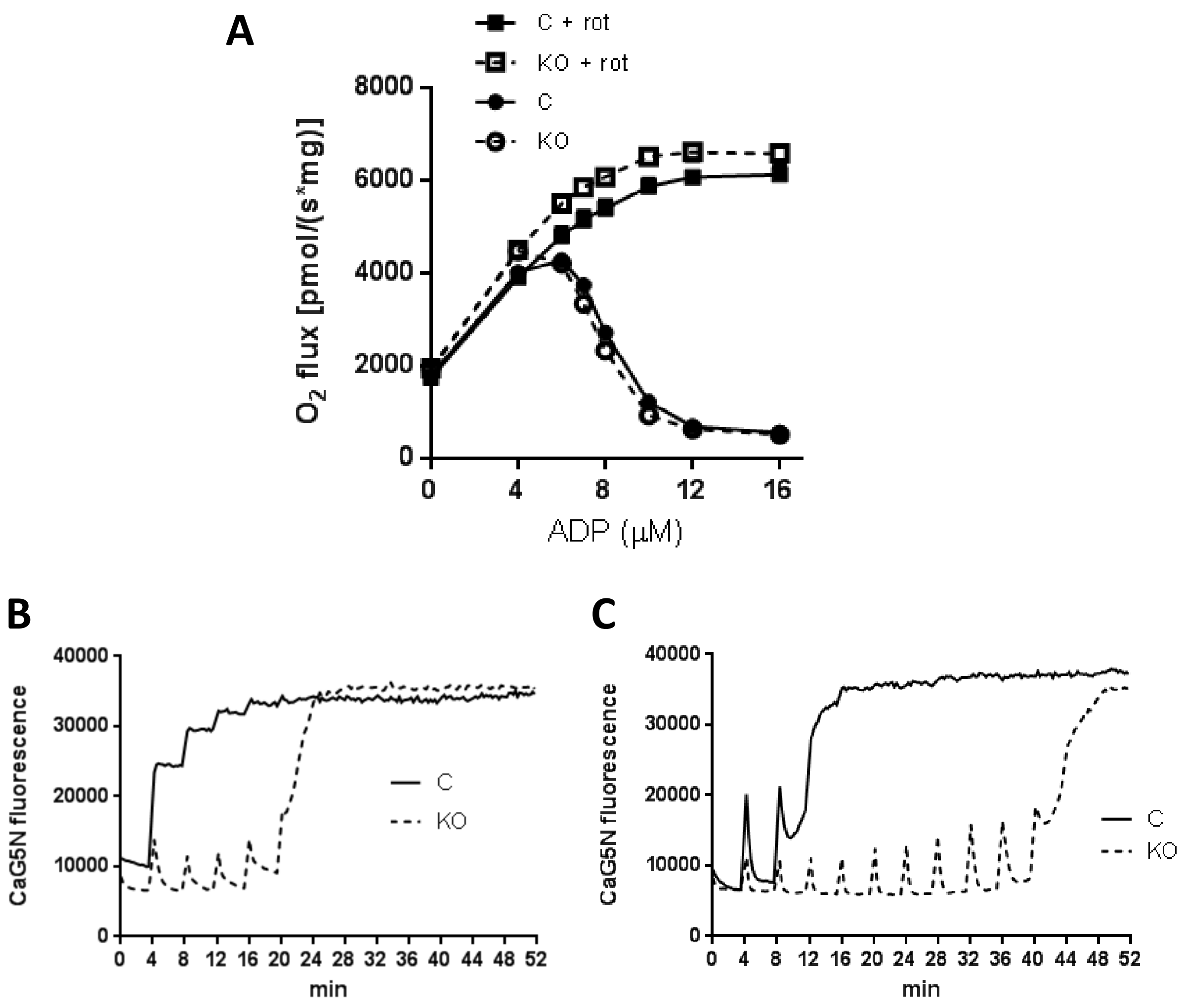

S1 Fig. Mitochondria respiring on succinate are not affected by the mitochondrial permeability transition pore. (A) O_2 flux determined in hindlimb muscle mitochondria of control (C) and CypD knock out (KO) mice respiring on succinate (5 mM) in the presence or absence of rotenone (rot) (5 µM) incubated as in the legend to figure 2 (main text) in the presence of HK and 2DOG. Data represent mean ± SE, n = 4 per group. No significant differences were observed between C and KO mice. (B) Calcium green fluorescence by mitochondria from a C and KO mouse. Mitochondria were energized by 5 mM succinate alone in the absence of ADP (state 4) with repeated administration of 5 µl of calcium chloride (455 µM) in 60 µl incubation volumes. (C) Incubations as in panel B, but in the presence of rotenone (5 µM). Panels B and C are representative of experiments repeated for all four C and KO mice using mitochondria from the preparations utilized for the experiments in panel A. Data show delayed opening of the MTP in KO mitochondria as manifest by greater Ca^{2+} retention, thus confirming the effectiveness of the KO perturbation. The difference in Ca^{2+} retention time between panels B and C (greater with rotenone) is an incidental finding but is compatible with the much greater ROS production at 0 ADP in the absence versus presence of rotenone (Fig 2F), even though potential did not differ at 0 ADP (Fig 2C).
